# Distinguishing *Myo*‐Inositol From Glycine in Brain MRS at 3T: A Pitfall Using Intermediate Echo Times

**DOI:** 10.1111/jon.70107

**Published:** 2025-11-11

**Authors:** Seyma Alcicek, Georg Oeltzschner, Doris D. M. Lin, Peter B. Barker

**Affiliations:** ^1^ Russell H. Morgan Department of Radiology and Radiological Science Johns Hopkins University School of Medicine Baltimore Maryland USA; ^2^ Goethe University Frankfurt, Institute of Neuroradiology and Cooperative Brain Imaging Center ‐ CoBIC Frankfurt am Main Germany; ^3^ F.M. Kirby Center for Functional Brain MRI, Kennedy Krieger Institute Baltimore Maryland USA

**Keywords:** brain, glycine, hypoxic‐ischemic encephalopathy, MR spectroscopy, *myo*‐inositol, nonketotic hyperglycinemia

## Abstract

**Background and Purpose:**

In in vivo magnetic resonance spectroscopy (MRS) of the brain, glycine (Gly) is traditionally separated from the overlapping signal of *myo*‐inositol (mI) through the use of intermediate (e.g., 130–140 ms) or long (270–280 ms) echo times (TE). However, no quantitative comparisons have been performed to date comparing the performance of clinically available MRS sequences to differentiate mI and Gly as a function of TE.

**Methods:**

In vivo spectra recorded with two clinically available MRS pulse sequences (single voxel PRESS and semi‐LASER 2D‐MRSI) with short (35 ms), intermediate (135 ms), and long (280 ms) echo times in a neonate with clinically suspected nonketotic hyperglycinemia were compared to those recorded from phantoms, and spectral simulations.

**Results:**

In vivo spectra recorded at short and intermediate TE spectra showed signals at 3.5 ppm that could arise from either mI or Gly; however, long TE spectra showed an absence of signal in this spectral region, which was consistent with the final clinical diagnosis of hypoxic‐ischemic encephalopathy. Phantom data and spectral simulations demonstrated that at intermediate TE, mI has a “pseudo‐singlet” appearance that is very similar to that of Gly.

**Conclusions:**

Long echo times are used to best discriminate Gly from mI if specialized sequences and analysis methods are not available. Quantitative spectral analysis methods may also assist in correctly assigning Gly and mI.

## 1 Introduction

In vivo magnetic resonance spectroscopy (MRS) of the human brain at 3T can measure approximately a dozen different metabolites [1]. Two of these compounds are glycine (Gly) and *myo*‐inositol (mI), which have a strong degree of overlap in the region of the spectrum around 3.55 ppm and, therefore, are often reported as a composite (sum) peak because of the difficulty in separating them. Despite the spectral overlap of mI and Gly, they have very different structures and functions. Gly is an amino acid with inhibitory neurotransmitter properties, acting as a neuromodulator of excitatory glutamatergic transmission as a co‐agonist of N‐methyl D‐aspartate receptors, while mI is a cyclic sugar alcohol often proposed to be a marker of glial cell proliferation and a regulator of osmotic balance. Gly is present at low concentrations (i.e., <1 mM) in normal brain [2] but increases in certain conditions, such as brain tumors [3 ^,^ 4], or nonketotic hyperglycinemia (NKH), a rare neurometabolic disorder caused by defects in the Gly cleavage enzyme system [5].

A variety of advanced MRS methods have been proposed to distinguish between mI and Gly; since mI has six inequivalent, coupled protons at a variety of chemical shifts ranging from 3.27 to 4.05 ppm [1], whereas Gly is a singlet at 3.55 ppm, advanced spectral fitting procedures (for instance, the “LCModel” [6]) may be able to separate them, particularly if spectral acquisition parameters are optimized to minimize overlap between basis spectra. For instance, it was shown that the 3.5 ppm mI signal was almost totally dephased (suppressed) by J‐coupling by using a customized “triple‐refocused” sequence at TE 198 ms, allowing the Gly peak to be clearly observed [2]. Other approaches used two‐dimensional J‐spectroscopy [7] or TE‐averaged PRESS at 4T [8] to separate Gly and mI; Gly has also been quantified at 7T using a short TE “SPECIAL” MRS pulse sequence [9].

However, none of these methods are routinely available on clinical 3T MR scanners. Therefore, typical clinical practice is to use intermediate (e.g., 130–140 ms) [5 ^,^ 10 ^,^ 11] or long (270–280 ms) TE MRS sequences in order to dephase the mI signal, and assume that any remaining signal results from Gly. In this short communication, the appearance of Gly and mI peaks in MR spectra acquired using vendor‐provided PRESS and semi‐LASER (sLASER) sequences at short (≤ 40 ms), intermediate (130–140 ms), and long TEs (270–280 ms) is examined using simulations and in phantoms, and in vivo in a neonatal patient. It is demonstrated that the pseudo‐singlet appearance of mI at intermediate TE can be misinterpreted as Gly, potentially leading to an erroneous diagnosis of hyperglycinemia.

## 2 Methods

### 2.1 MRI and MRS Protocol

MRS data were acquired on a 3T “Skyra” scanner equipped with a 20‐channel receive head coil (Siemens Healthineers, Erlangen, Germany). The neonatal brain magnetic resonance imaging (MRI) protocol included a variety of sequences, including axial T_2_ (TR 5.16 s, TE 93 ms, 43 slices, voxel size 1×1×3 mm^3^) and DTI (TR 9.7 s, TE 86 ms, 55 slices, voxel size 2×2×2 mm^3^, b = 1000 s/mm^2^).

SV PRESS MRS was performed at TE of 35 and 280 ms (TR = 1.5–2 s, number of transients = 80–192, voxel size = 15×15×20 mm^3^) in the bilateral thalamus and right frontal white matter. These voxel locations are standard for the neonatal MRS protocol, and are chosen because they give good technical quality spectra and are often involved in the pathological processes that occur in neonates (e.g., hypoxic‐ischemic encephalopathy [HIE]—deep gray matter and watershed regions). Multi‐voxel sLASER was performed at TE of 40 and 135 ms (TR = 2 s, number of transients = 3, field of view = 160×160 mm^2^, slice thickness = 15 mm, matrix size = 16×16, nominal voxel size = 10×10×15 mm^3^) in the axial plane at the level of the thalamus. Voxels from the thalamus and white matter regions were selected for further evaluation. Note that because of time limitations associated with the scan performed for clinical purposes, it was not possible to record both SV and MRSI data at all three echo times (i.e., short, intermediate, and long).

### 2.2 Phantom Data

A phantom (GE “braino”) containing 12.5 mM N‐acetyl aspartate, 10 mM creatine, 3 mM choline, 7.5 mM mI, 5 mM Lac, and 12.5 mM glutamate was measured at room temperature with vendor‐provided single‐voxel (SV) PRESS MRS at TE of 35, 135, and 280 ms, and multi‐voxel sLASER‐MRSI at TE of 40, 135, and 270 ms, with other parameters similar to the in vivo acquisitions.

### 2.3 Spectral Analysis

In addition to Gly and mI, the following compounds were included in the basis sets for fitting of in vivo and phantom spectra: ascorbate, aspartate, creatine, ethanolamine, γ‐aminobutyric acid, glycerophosphocholine, glutathione, glutamine, glutamate, lactate, N‐acetylaspartate, N‐acetylaspartyl glutamate, phosphocholine, phosphocreatine, phosphoethanolamine, scyllo‐inositol, and taurine. Chemical shifts and coupling constants were taken from the literature [1].

Spectral simulations were performed employing MRSCloud [12], assuming ideal excitation, with sequence timings and refocusing RF pulse waveforms used in PRESS and sLASER sequences at short (35 and 40 ms, respectively), intermediate (135 ms), and long TE (270 and 280 ms). MRS data were analyzed using the “Osprey” [13] (Baltimore, MD, USA, https://github.com/schorschinho/osprey) open‐source analysis toolbox with the integrated LCModel (v.6.3‐1N,  Oakville, Ontario, Canada, https://s‐provencher.com/lcmodel.shtml) fitting algorithm binary for fitting [14]. T_1_ and T_2_ relaxation time corrections were not performed.

The ability to differentiate Gly from mI across different TEs was estimated using LCModel coefficients of model covariance (CMC), a metric derived from the Fisher information matrix that quantifies the interaction between two model parameters within a single dataset. Cramér−Rao lower bounds (CRLBs) were also reported for mI and Gly.

## 3 Results

A term male infant with suspected HIE after placental abruption and fetal anemia of unclear etiology was placed on therapeutic hypothermia. There was significant acidosis on cord gas, and the initial Sarnat exam revealed moderate encephalopathy at 3 h of life. On exam, he was very hypotonic with minimal reactivity. History and initial perinatal course were highly suspicious for HIE, but neurological exam and seizures on cranial electroencephalography were out of proportion to imaging findings on ultrasound, on which there was no evidence of edema. HIE mimics were considered, including epileptic encephalopathies such as NKH or Ohtahara syndrome. The patient was referred for a brain MRI and MRS.

**FIGURE 1:**
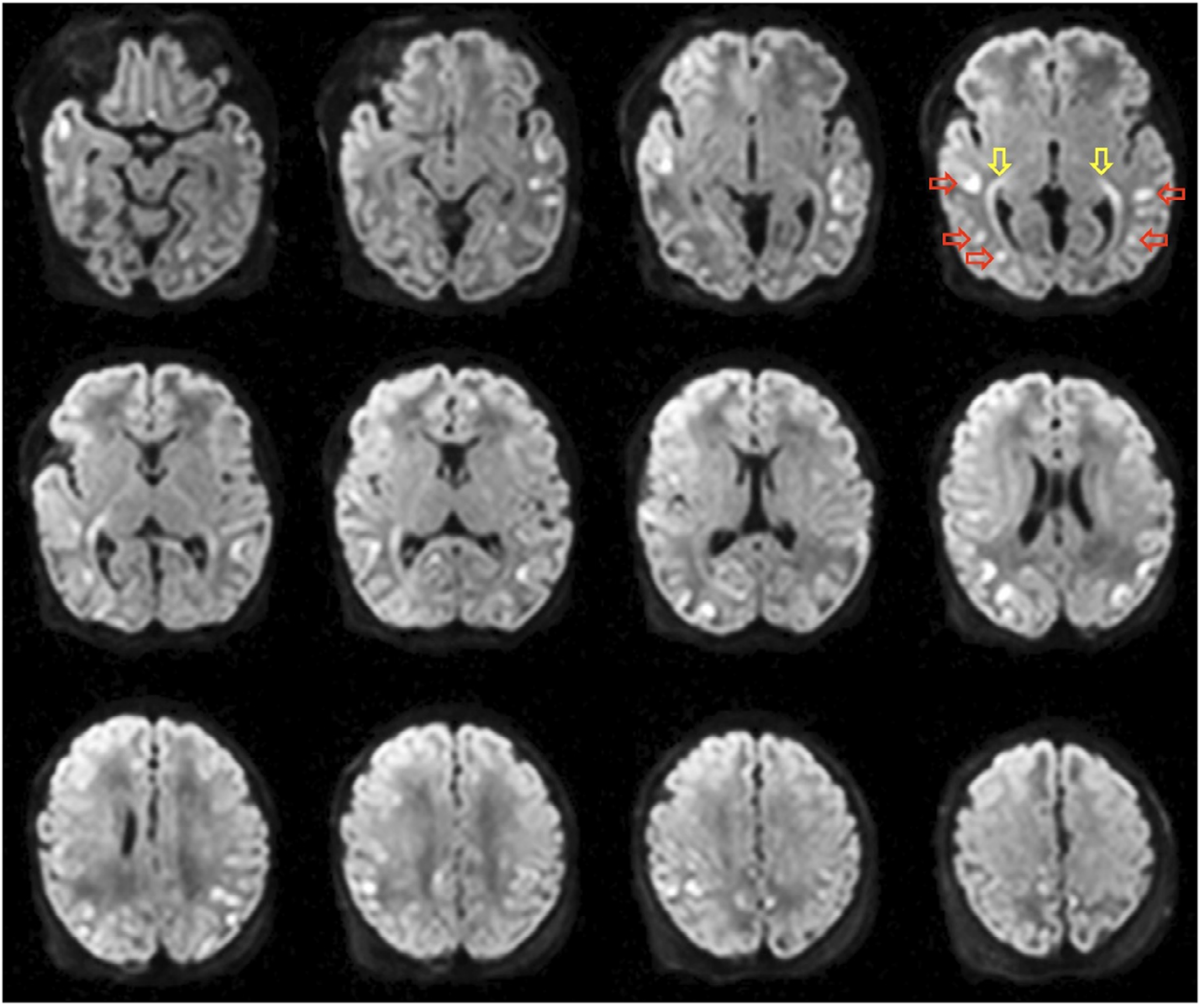
Diffusion‐weighted images at 6 days of age show multiple cortical foci of restricted diffusion (illustrated on slice 4, red horizontal arrows), also involving the retrolenticular segments of the internal capsule and the posterior thalamic radiations (yellow vertical arrows).

**FIGURE 2:**
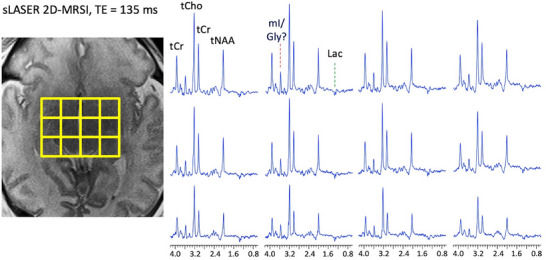
Semi‐LASER 2D magnetic resonance spectroscopic imaging data recorded at intermediate echo time (TE 135 ms). The central 4×3 voxels (nominal voxel size is 10×10×15 mm^3^) of the 16×16 matrix are plotted. Peaks are assigned to total choline (tCho), total creatine (tCr), total N‐acetylaspartate (tNAA), and lactate (Lac, an inverted signal at this TE). There is a prominent singlet peak at 3.55 ppm in all voxels that was initially assigned to glycine (Gly), but subsequent investigations (see text and Figures 4 and 5) suggest that this most likely originates from *myo*‐inositol (mI).

**FIGURE 3:**
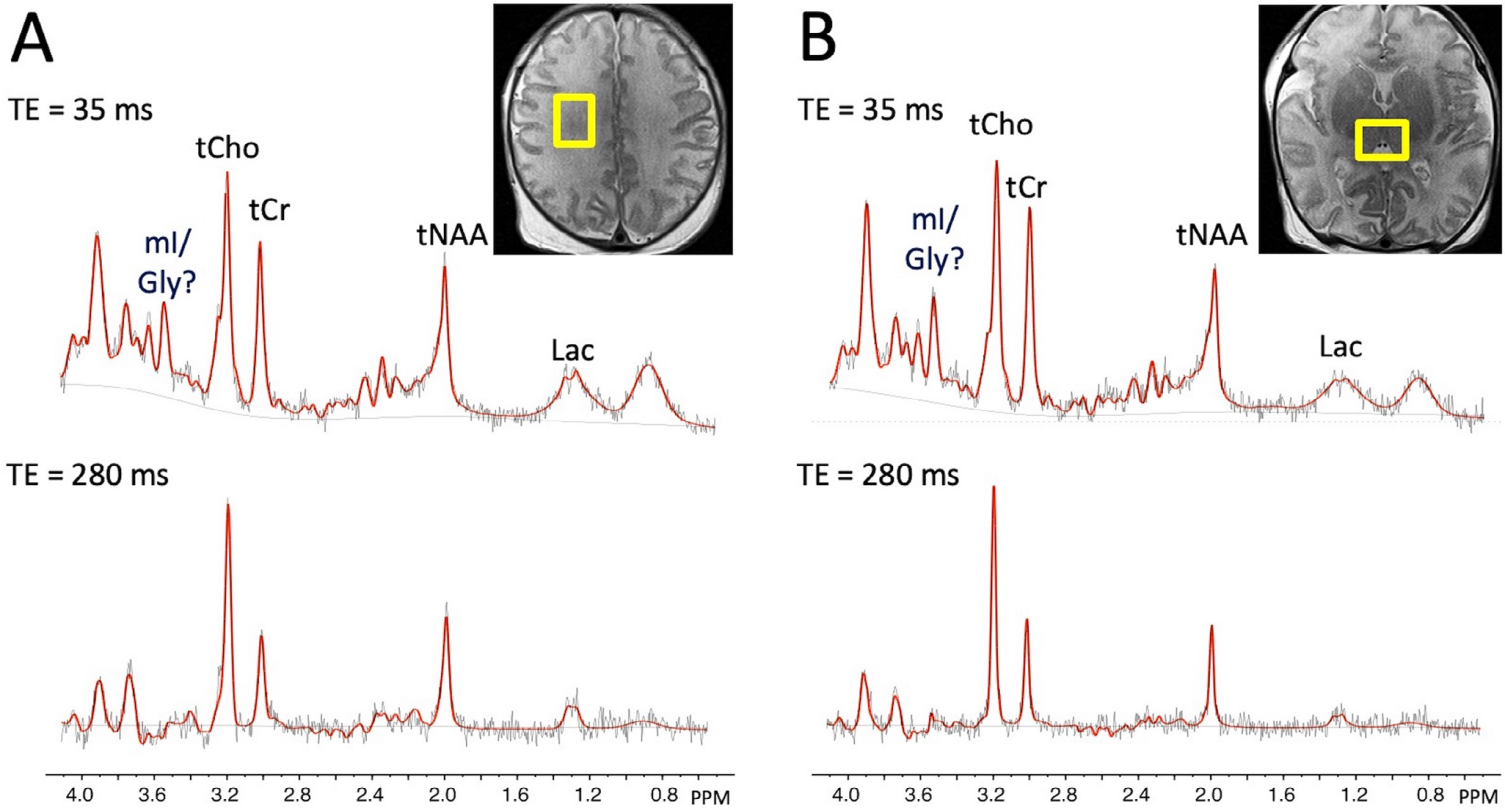
Point‐resolved spectroscopy sequence (PRESS) localized single voxel spectra recorded from (A) right frontal white matter and (B) bilateral thalamus at both short (TE 35 ms) and long (TE 280 ms) echo times. Voxel size was 15×20×15 mm^3^ and voxel locations are indicated in yellow superimposed on T_2_‐weighted MRI. Peak assignments are total choline (tCho), total creatine (tCr), total N‐acetylaspartate (tNAA), glycine (Gly), *myo*‐inositol (mI), and lactate (Lac). No clear peak is visualized at 3.55 ppm in either brain region in the long TE spectra. Black lines are the original data, and red lines are the result of LCModel fitting.

On MRI, multiple foci of restricted diffusion were noted (Figure 1). Multi‐voxel sLASER data at short (TE 40 ms) and intermediate echo times (TE 135 ms) are shown in Figure 2. SV PRESS localized MRS performed at short (TE 35 ms) and long (TE 280 ms) echo times are shown in Figure 3.

**FIGURE 4:**
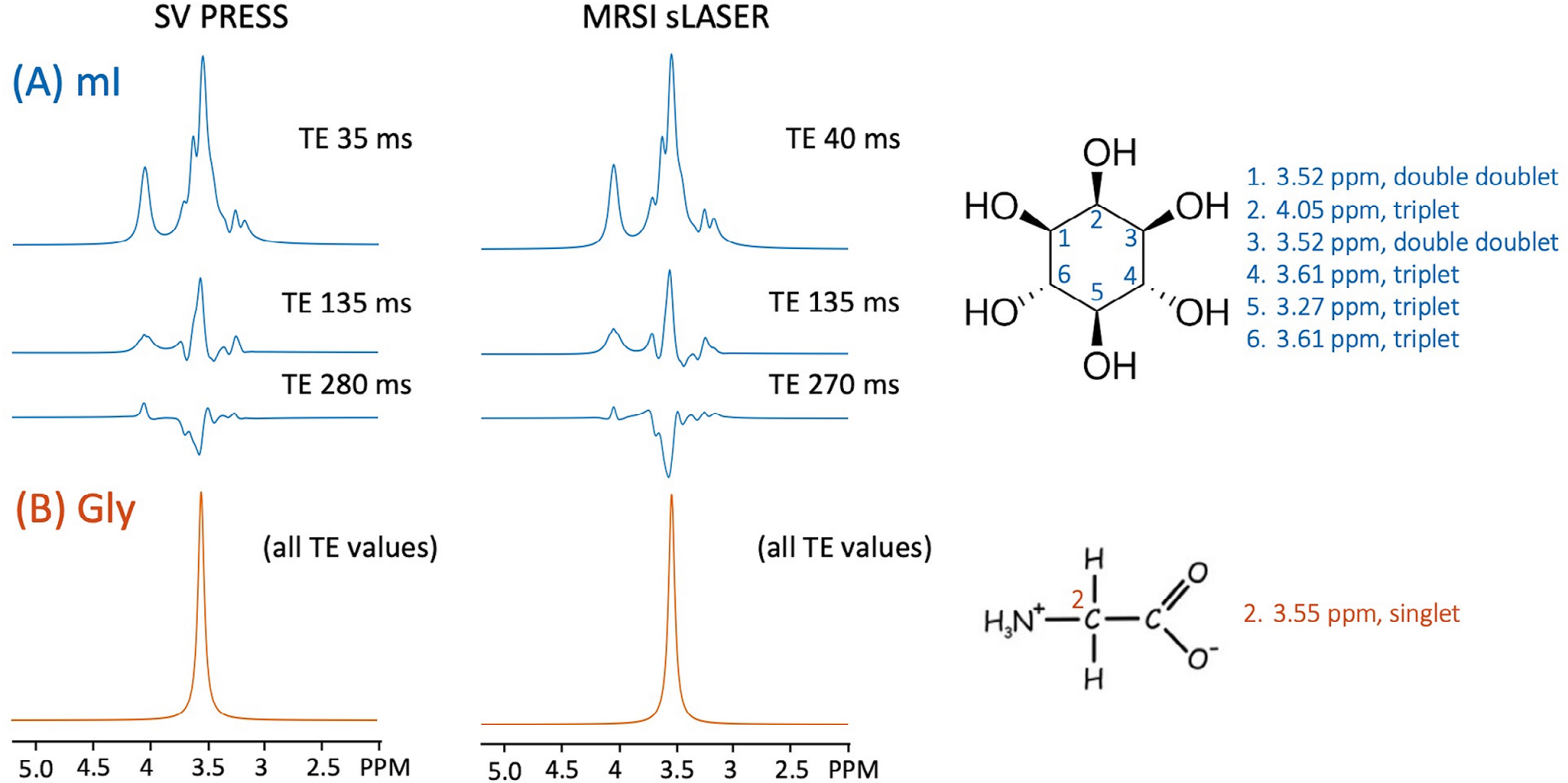
3T spectral simulations (with no T_2_ decay, line width = 8 Hz) plotted between 2.0 and 5.2 ppm for (A) *myo*‐inositol (mI) and (B) glycine (Gly) for the single voxel point‐resolved spectroscopy (PRESS) sequence at TE 35, 135, and 280 ms and for the magnetic resonance spectroscopic imaging semi‐LASER (sLASER) sequence at TE 40, 135, and 270 ms. Gly is a singlet at all TE values, whereas the mI multiplets vary considerably as a function of TE; similar mI patterns are seen for PRESS and sLASER, although signal intensity is slightly higher for sLASER at longer TE values. Also shown are the molecular structures and peak assignments for both mI and Gly.

**FIGURE 5:**
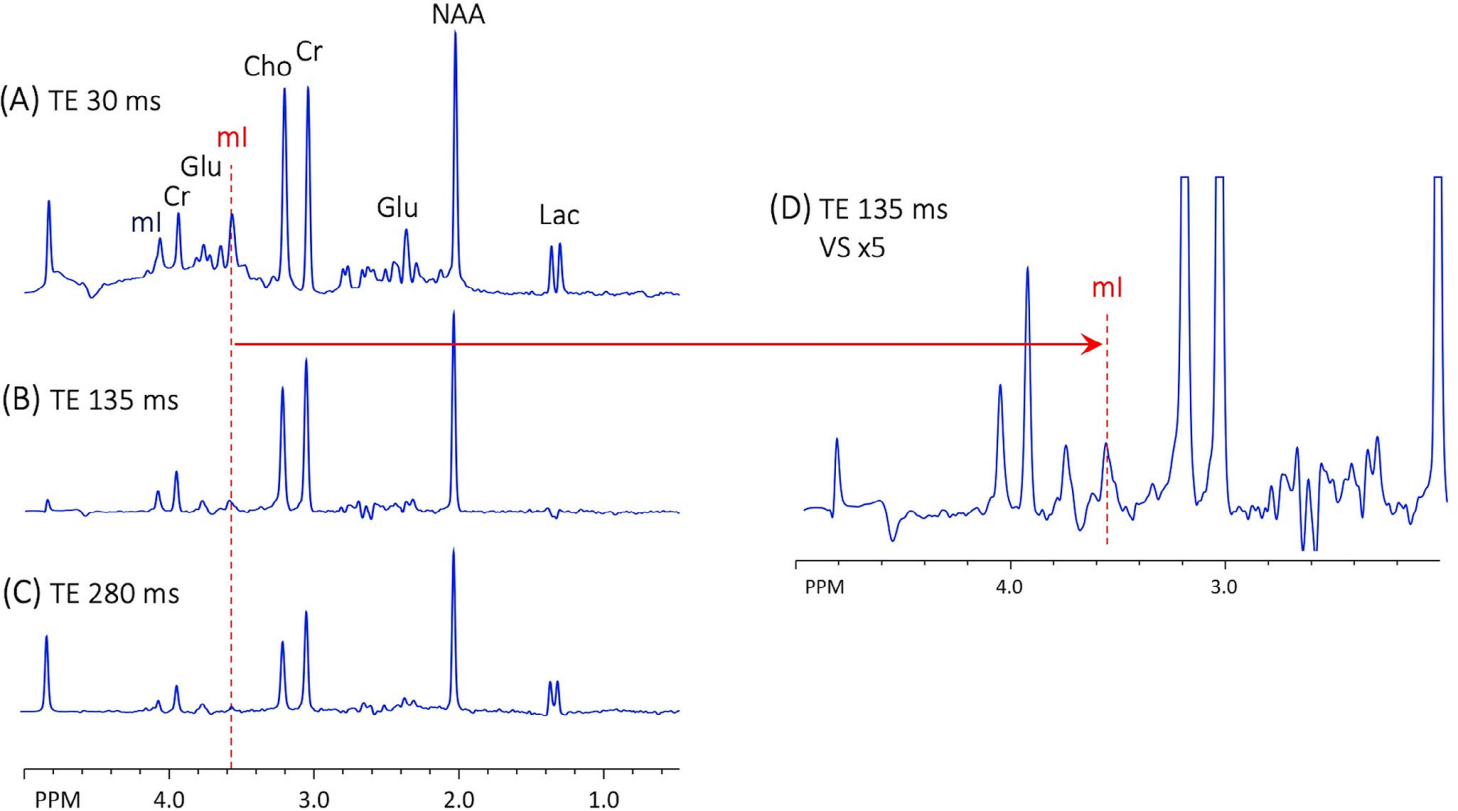
3T single voxel point‐resolved spectroscopy of the GE “braino” phantom which contains *myo*‐inositol (mI) (but no glycine) at (A) TE 30, (B) 135, and (C) 280 ms; also shown in (D) is the TE 135 ms spectrum with the vertical scale (VS) increased by a factor of 5. Peaks are assigned to lactate (Lac), N‐acetylaspartate (NAA), glutamate (Glu), creatine (Cr), and choline (Cho).

In the intermediate TE sLASER 2D‐MRSI spectra, there was a prominent singlet peak at 3.55 ppm throughout the dataset that was initially interpreted as being attributable to Gly (Figure 2). Otherwise, MRSI showed near normal levels of total choline (tCho), total creatine (tCr), and total N‐acetylaspartate (tNAA) for a 6‐day‐old infant, with a small elevation of lactate (Lac). SV MRS of the thalamus and right frontal white matter showed similar spectral patterns as on 2D‐MRSI; however, there was no obvious Gly signal at 3.55 ppm in the long TE spectra (Figure 3). Subsequent spectral simulations (Figure 4) and spectra recorded in a phantom containing mI (Figure 5) confirmed that the 3.55 ppm peak seen in the brain at intermediate TE originates from mI, not Gly. Genetic testing for NKH was negative, and the final diagnosis based on MRI and clinical exam was mild HIE.

Quantitative LCModel results of spectra recorded at short, intermediate, and long TE, and associated CRLB and CMC values are given in Table 1. At short TE, the LCModel reported relatively low CRLB values (e.g., < 25% or better) for both mI and Gly; however, the ratio of mI/Gly was lower than literature values (i.e., in the range 2.2−3.5), compared to an average of around 9 in the literature (Table 2), and there were strong negative CMC values (−0.63 to −0.75) indicating a high degree of similarity between basis functions. Conversely, at TE 135 ms, LCModel only identified a mI peak, but no Gly, while at TE 280 ms, generally higher CRLB values were found for both mI and Gly, reflecting signal decay, and also positive CMC values (0.46−0.55) indicating a positive relationship between concentrations (because the signals in the basis functions at this TE are of opposite sign). Overall, the high CMC values for mI and Gly, regardless of TE, and the variability of the mI/Gly ratio, suggest that LCModel results from nonoptimized MRS/MRSI sequences should be interpreted with caution when trying to quantify mI and Gly separately.

**TABLE 1:** Results of LCModel analysis from the thalamus and frontal white matter with different pulse sequences and echo times.

	mI/tCr	CRLB	Gly/tCr	CRLB	mI/Gly	mI/Gly CMC values	Lac/tCr	CRLB
SV PRESS								
Thalamus TE 35 ms	0.63	9%	0.29	16%	2.21	−0.75	0.04	121%
Thalamus TE 280 ms	1.11	19%	0.24	21%	4.53	0.46	0.08	95%
Frontal WM TE 35 ms	0.82	9%	0.23	24%	3.56	−0.74	0.15	41%
Frontal WM TE 280 ms	1.48	20%	0.16	48%	9.12	0.55	0.18	71%
sLASER MRSI								
Thalamus TE 40 ms	1.15	5%	0.46	10%	2.49	−0.63	0.24	14%
	1.29	6%	0.52	11%	2.47	−0.73	0.41	13%
Thalamus TE 135 ms	1.74	6%	0.00	—	—	—	0.36	9%
	1.92	6%	0.00	—	—	—	0.64	14%

Abbreviations: CMC, coefficients of model covariance; CRLB, Cramer–Rao lower bounds; Gly, glycine; Lac, lactate; MRSI, magnetic resonance spectroscopic imaging; ms, millisecond; PRESS, point‐resolved spectroscopy sequence; sLASER, semi‐LASER; SV, single voxel; tCr, total creatine; TE, echo times; WM, white matter.

**TABLE 2:** Literature values of brain mI and Gly values determined by MRS.

Reference	Year	Sequence	TE (ms)	Voxel location	Population	B_0_	[mI]	mI/tCr	[Gly]	Gly/tCr	mI/Gly
Gray matter											
Schulte and Boesiger [7]	2006	2DJ	31−229	Parietal lobe	HV	3T		0.99		0.12	8.0
Prescot et al. [8]	2006	TEavg	30−284	Occipital cortex	HV	4T	—			0.03	—
Choi et al. [2]	2008	Triple_ref	198	Parieto‐occipital cortex	HV	3T	5.80		0.50		11.6
Gambarota et al. [9]	2009	SPECIAL	30	Occipital lobe	HV	7T		0.61		0.14	4.4
Banerjee et al. [15]	2012	PRESS	150	Frontal and parietal lobes	HV	7T	6.90		1.10		6.3
Marjanska et al. [16]	2012	LASER	35	Occipital lobe	HV	7T	6.40		0.70		9.1
Li et al. [17]	2015	MRSI	30	NS	Glioma	7T		0.61		0.17	3.6
Ganji et al. [18]	2016	MRSI	160	Mesial frontal lobe	HV/glioma^a^	3T	10.20		1.10		9.3
Murali‐Manohar et al. [19]	2020	semi‐LASER	24−60	Occipital lobe	HV	9.4T	5.22		1.11		4.7
Average							6.90	0.74	0.90	0.12	7.1
Standard deviation							1.95	0.22	0.28	0.06	2.8
White matter											
Hofmann et al. [20]	2002	PRESS	20	CSO	HV	1.5T	4.53		0.48		9.4
Banerjee et al. [15]	2012	PRESS	150	Frontal and parietal lobes	HV	7T	1.60		0.10		16.0
Li et al. [17]	2015	MRSI	30	NS	Glioma	7T		0.66		0.20	3.3
Ganji et al. [18]	2016	MRSI	160	Frontal lobe	HV/glioma	3T	5.70		0.30		19.0
Average							3.94		0.29		11.9
Standard deviation							2.11		0.19		7.0

*Note*: In studies involving glioma patients, results are reported from normal appearing regions on MRI.

Abbreviations: 2DJ, two‐dimensional J‐resolved spectroscopy; CSO, centrum semiovale white matter; Gly, glycine; HV, healthy adult volunteers; LASER, localization by adiabatic selective refocusing; mI, *myo*‐inositol; MRSI, magnetic resonance spectroscopic imaging; NS, not specified; PRESS, point‐resolved spectroscopy sequence; SPECIAL, spin echo full‐intensity acquired localization; T, Tesla; tCr, total creatine; TEavg, TE‐averaged PRESS; Triple_ref, triple‐refocused spectroscopy sequence.

^a^Results reported from HV only here.

## 4 Discussion

This study demonstrates that 3T MRSI at intermediate TEs (e.g., 130–140 ms) shows similar signals from mI and Gly; therefore, they cannot be distinguished from each other by visual inspection alone—in particular, mI has a “pseudo‐singlet” appearance that quite closely matches the appearance and chemical shift of the Gly peak. At longer TE values (e.g., 280 ms), most of the mI signal has dephased due to J‐modulation, so its spectrum no longer has a visual appearance that mimics Gly (Figure 4). The simulations in Figure 4 indicate that at long TE (i.e., 270–280 ms), the 3.55 ppm mI signal is about a factor of 5 smaller than Gly (and has opposite polarity), assuming no difference in T_2_ relaxation times. As noted in the introduction, more advanced sequences (and or the use of higher magnetic field strengths, such as 7T) are available for mI/Gly discrimination, but are typically not commercially available on clinical MR scanners. Similarly, quantitative LCModel analysis of data recorded using commercially available sequences may also be helpful in distinguishing mI from Gly, but accuracy depends strongly on acquisition parameters and spectral quality.

Misinterpretation of mI as Gly could have important implications in certain clinical scenarios; for instance, it might lead to an erroneous diagnosis of NKH, which then could potentially lead to unnecessary clinical follow‐up and even inappropriate management [21]. The other main scenario where Gly may have clinical significance is in the evaluation of brain tumors, where higher Gly levels have been reported in high‐grade compared to low‐grade glioma [18 ^,^ 22], although this was not observed in one other study [3]. Nevertheless, an in vitro metabolic profiling study did show that glycine consumption and expression of the mitochondrial glycine biosynthetic pathway were strongly correlated with cancer cell proliferation [23].

For all resonances, T_2_‐related signal decay occurs as TE increases; in addition, for coupled spin systems such as mI, multiplet patterns will change due to the evolution of scalar (J) couplings. There have been a few reports of T_2_ values for mI and Gly in the human brain, although one study at 3T did report very similar T_2_ relaxation times for Gly and mI in white matter (152 and 161 ms, respectively), with more diverging values for cortical regions [24]. A study at the high magnetic field strength of 9.4T did report slightly higher T_2_ values for mI compared to Gly (90 ± 18 vs. 61 ± 11 ms, respectively) [19]. Note, however, that it is very difficult to measure mI and Gly T_2_ values for multiple reasons, including their high degree of spectral overlap at low TE values, the low Gly concentration in normal brain, J‐modulation effects on the mI signal, and also contributions from underlying macromolecular resonances [19 ^,^ 24 ^,^ 25]. Nevertheless, if Gly does have a significantly lower T_2_ than mI, this will reduce the effectiveness of long TE acquisitions to selectively detect Gly.

Two different commercially available pulse sequences were used in this study: SV PRESS and sLASER‐based 2D‐MRSI. While simulations (Figure 4) showed that the mI signal as a function of TE showed similar behavior between the two sequences, in detail, there are significant differences in evolution (in particular, slightly higher mI signal intensities for sLASER at intermediate or long TE) reflecting both the differences between SV and MRSI localization, as well as the different pulse sequences. sLASER uses pairs of adiabatic pulses for refocusing, which have high bandwidths and excellent slice profiles. In addition, for sLASER‐MRSI, a large volume of interest is excited by the localization sequence, so the signal loss due to sub‐voxel compartmentation effects [26] encountered in SV MRS is not an issue for MRSI [27].

Finally, it should be noted that the ratio of mI/Gly determined by LCModel analysis in vivo varied from 2.2 to 9.1 (Table 1), depending on the brain region and sequence parameters used. Given this wide variation in the mI/Gly ratio, Gly levels estimated from LCModel analysis of MRS data acquired with “nonoptimized” pulse sequences should be interpreted with caution.

In conclusion, in 3T MRS, the mI spectrum at intermediate TE (135–140 ms) exhibits a pseudo‐singlet signal at ∼3.55 ppm, mimicking Gly and potentially causing mis‐assignment. For intermediate TE, linear‐combination modeling appears to be required, while visual assessment may be possible at longer TEs (270–280 ms) to validate the presence of elevated Gly levels. However, the use of long TEs also results in lower spectral SNR due to T_2_ relaxation time effects. Incorporating fitting methods such as the LCModel into clinical spectral evaluation, alongside visual assessment, may be beneficial to avoid misinterpretation.

## Funding

The study was supported in part by NIH P41EB031771, R01EB028259, R01NS134694, R21EB033516, and R01EB035529.

## Conflicts of Interest

No relevant conflicts of interest.
